# Towards crystal structure prediction of complex organic compounds – a report on the fifth blind test

**DOI:** 10.1107/S0108768111042868

**Published:** 2011-11-17

**Authors:** David A. Bardwell, Claire S. Adjiman, Yelena A. Arnautova, Ekaterina Bartashevich, Stephan X. M. Boerrigter, Doris E. Braun, Aurora J. Cruz-Cabeza, Graeme M. Day, Raffaele G. Della Valle, Gautam R. Desiraju, Bouke P. van Eijck, Julio C. Facelli, Marta B. Ferraro, Damian Grillo, Matthew Habgood, Detlef W. M. Hofmann, Fridolin Hofmann, K. V. Jovan Jose, Panagiotis G. Karamertzanis, Andrei V. Kazantsev, John Kendrick, Liudmila N. Kuleshova, Frank J. J. Leusen, Andrey V. Maleev, Alston J. Misquitta, Sharmarke Mohamed, Richard J. Needs, Marcus A. Neumann, Denis Nikylov, Anita M. Orendt, Rumpa Pal, Constantinos C. Pantelides, Chris J. Pickard, Louise S. Price, Sarah L. Price, Harold A. Scheraga, Jacco van de Streek, Tejender S. Thakur, Siddharth Tiwari, Elisabetta Venuti, Ilia K. Zhitkov

**Affiliations:** a Cambridge Crystallographic Data Centre, 12 Union Road, Cambridge CB2 1EZ, England; b Imperial College London, England; c Cornell University, USA; d South Ural State University, Russian Federation; e SSCI, An Aptuit Company, USA; f Department of Chemistry, University College London, England; g The Pfizer Institute for Pharmaceutical Materials Science, University Chemical Laboratory, University of Cambridge, England; h University of Amsterdam, The Netherlands; i Department of Chemistry, University of Cambridge, England; j Università di Bologna, Italy; k Indian Institute of Science, India; l University of Utrecht, The Netherlands; m Center for High Performance Computing, University of Utah, USA; n Department of Biomedical Informatics, University of Utah, USA; o Universidad de Buenos Aires, Argentina; p Parco Scientifico e Technologico, Italy; q FlexCryst, Germany; r University Erlangen–Nürnberg, Germany; s Ruhr-Universität Bochum, Germany; t University of Bradford, England; u Vladimir State Humanitarian University, Russian Federation; v Cavendish Laboratory, England; w Avant-garde Materials Simulation, Germany; x Department of Physics and Astronomy, University College London, England

**Keywords:** prediction, blind test, polymorph, crystal structure prediction

## Abstract

Following on from the success of the previous crystal structure prediction blind tests (CSP1999, CSP2001, CSP2004 and CSP2007), a fifth such collaborative project (CSP2010) was organized at the Cambridge Crystallographic Data Centre. A range of methodologies was used by the participating groups in order to evaluate the ability of the current computational methods to predict the crystal structures of the six organic molecules chosen as targets for this blind test. The first four targets, two rigid molecules, one semi-flexible molecule and a 1:1 salt, matched the criteria for the targets from CSP2007, while the last two targets belonged to two new challenging categories – a larger, much more flexible molecule and a hydrate with more than one polymorph. Each group submitted three predictions for each target it attempted. There was at least one successful prediction for each target, and two groups were able to successfully predict the structure of the large flexible molecule as their first place submission. The results show that while not as many groups successfully predicted the structures of the three smallest molecules as in CSP2007, there is now evidence that methodologies such as dispersion-corrected density functional theory (DFT-D) are able to reliably do so. The results also highlight the many challenges posed by more complex systems and show that there are still issues to be overcome.

## Abstract 2

The results of the fifth blind test of crystal structure prediction, which show important success with more challenging large and flexible molecules, are presented and discussed.

## 1. Introduction

This paper reports on the results of the fifth blind test of crystal structure prediction (CSP), an international test hosted periodically by the Cambridge Crystallographic Data Centre (CCDC). We refer to this fifth blind test as CSP2010.

Over the last several decades there has been much research in the field of crystal structure prediction. The grand aim is to develop the ability to reliably predict, by computational methods, how a molecule will crystallize in the solid state, with only the chemical diagram and the crystallization conditions known. This would allow for the prediction of solid-state properties before the molecule or molecules in question had even been synthesized, and could also help determine the likelihood that different polymorphic forms, or as yet unseen polymorphs of currently known structures, exist. This application is of particular importance in the pharmaceutical industry where the presence of different polymorphs can lead to very different and potentially undesirable physical properties of new drugs.

For the last decade the CCDC has held periodic blind tests to assess the current reliability and capabilities of the techniques available in the field. Four blind tests, starting in 1999 and every 2 or 3 years thereafter, have previously been held. Each has required the identification of a set of molecules with known but previously unpublished crystal structures to use as targets for the participants to predict using the various techniques they have developed. This approach is similar to that adopted to monitor and test advances in other areas of predictive modelling, such as protein structure prediction (Moult *et al.*, 2007 ▶). Recently there has also been a blind test for search methods for the crystal structure prediction of purely inorganic systems (Oganov, 2010 ▶). Repeating the blind test periodically helps to evaluate advances that have been made in methodologies since the last test, as well as establish the reliability of the techniques which have been successful in previous tests for a given category of target; the small number of targets in any one blind test introduces the possibility of a slightly easier or harder molecule (whose difficulty cannot be easily judged prior to commencement of the test) influencing the results.

This fifth blind test was therefore held to assess the reproducibility of the good results (Neumann *et al.*, 2008 ▶; Day *et al.*, 2009 ▶) from the previous blind test, CSP2007, and also to assess the developments in methodologies when applied to more challenging targets than the relatively simple rigid molecules mostly studied thus far. These additional targets better represent cases that would be more likely to be encountered in the pharmaceutical industry.

## 2. Organization and approach

The organization for this latest blind test, CSP2010, was similar to that used for the previous four evaluations of the field, the results of which have been previously published: CSP1999 (Lommerse *et al.*, 2000 ▶), CSP2001 (Motherwell *et al.*, 2002 ▶), CSP2004 (Day *et al.*, 2005 ▶) and CSP2007 (Day *et al.*, 2009 ▶). Invitations to participate were sent to 24 research groups known to be active in the field. The test was also advertised through various websites and meetings.

The previous blind test puts forward targets for prediction in the following four categories:

(1) Small, rigid molecules; only the elements C, H, N and O; *Z*′ = 1 in any space group; up to 25 atoms.
(2) Rigid molecules; unusual functional groups or elements such as halogens, S, P and B; *Z*′ = 1 in any space group; up to 30 atoms.
(3) Moderately flexible molecule with 2–4 internal degrees of freedom; *Z*′ = 1 in any space group; up to 40 atoms.
(4) Multiple independent rigid molecules, *e.g.* solvates, co-crystals, salts or *Z*′ = 2 structures; any space group; up to 30 atoms.

These four categories were left the same as those used in CSP2007 so as to facilitate comparison of results. In addition, it was decided to add two new categories that would provide greater challenges:

(5) Molecule with 4–8 internal degrees of freedom; *Z*′ ≤ 2 in any space group; 50–60 atoms.
(6) Molecule for which more than one polymorph is known, and which roughly falls into one of the first four categories.

The new fifth category presents a much greater challenge in terms of flexibility than previously encountered in earlier blind tests, with a large flexible molecule intended to represent those often associated with modern pharmaceuticals. The new sixth category gives an opportunity to study the challenging effects of polymorphism by introducing a molecule for which more than one polymorph is known.

Crystallographers were contacted in August 2009 with a request for unpublished crystal structures that matched one or more of the six categories for the fifth blind test. Crystal structures were collected at the CCDC and assessed for the possibility of inclusion in one of the six possible categories. To be suitable, a crystal structure had to be of high quality and have all atoms located with no disorder. The crystal structure had to be unpublished and the donor crystallographer had to agree to postpone any publication for the duration of the blind test. Collection of suitable candidates for all six categories proved exceptionally difficult, especially for category 1, where the target molecule is very small with a very restricted set of constituent elements, and also for category 6 where few suitable candidates were available that were not of sufficient interest to be withheld from publication for the duration of this test. Almost 30 submitted crystal structures had to be rejected either due to not conforming to any of the six categories, or the presence of refinement issues such as disorder.

After considerable effort, one candidate was collected for category 1, four for category 2, eight for category 3, three for category 4, three for category 5 and one for category 6. For those categories where there was more than one candidate, the final target choice was made randomly.

For category 6, the one candidate that was submitted was gallic acid monohydrate, for which two new polymorphs had been found. These complemented the two previously published polymorphs for gallic acid monohydrate, which are located in the Cambridge Structural Database (CSD; Allen, 2002 ▶) under the KONTIQ CSD reference code family. For the purposes of this blind test, these known forms are referred to as forms (1) and (2). Of the two new forms submitted as candidates for prediction, one [form (4), as recently published by Clarke *et al.*, 2011 ▶] had one formula unit in the asymmetric unit (*i.e.* one gallic acid and one water molecule). The other, form (3), was originally solved with two formula units per asymmetric unit. However, analysis after the blind test submissions showed that this solution contained a disordered hydrogen-bonding network and the crystal structure could also be described with an ordered hydrogen-bonding network by doubling the unit cell, as now published (Clarke *et al.*, 2011 ▶). For the purposes of this blind test, form (3) was therefore deemed inappropriate as a target crystal structure. The main aim for this category, then, was to predict form (4), whose structure has been recently independently published (Demirtaş *et al.*, 2011 ▶) and see where (if at all) forms (1) and (2) appeared in the ranked list of predictions.

The molecular diagrams and crystallization conditions were sent by e-mail to 15 participant groups on 16 November 2009. Immediately after circulation of the target crystal structures we were made aware that the crystal structure of the molecule selected for category 1 (4-ethynylbenzonitrile) had been solved, was undergoing publication and so would soon be in the public domain. The decision was therefore made to remove this candidate for category 1 and attempt to locate a suitable replacement. Thankfully a suitable candidate was quickly provided and the revised list of target molecules, as detailed in Table 1 ▶, was distributed to participants on 23 November 2009. Following the numbering used in the previous blind tests we refer to these molecules by the Roman numerals (XVI)–(XXI).

The format of this blind test was kept broadly the same as the last blind test, with the exception that a greater length of time was allowed before submission of results. Participants were requested to forward their three ‘official’ predictions for each target molecule to the CCDC, where the experimentally determined crystal structures were held for the duration of the test. As well as these three main predictions, participants were urged to submit an extended list of the crystal structures they generated in order to help post-analysis and to provide insight into the performance of the various methods. The deadline for submissions was 20 August 2010. The experimentally determined crystal structures for all six categories were then circulated to all participants on 23 August 2010 to allow post-analysis of their predictions. Lastly, a workshop was held at the CCDC mid-September 2010 to discuss the results.

We present here results from the 14 participating groups that agreed to publish their results. Details of these 14 participating groups, together with a summary of which targets they attempted and if a match with the experimental structure was observed in their submission, are presented in Table 2 ▶(*a*).

## 3. Methodologies

Methodologies for the participating research groups vary significantly. A summary of the techniques used by each of the groups is presented in Table 2 ▶(*b*), together with key references for most of the methods used. More detailed descriptions are also provided in the associated supplementary material.^1^

In general, each of the methods employed involved three general steps:

(i) building a three-dimensional molecular structure from the supplied two-dimensional chemical diagrams;
(ii) searching for plausible crystal packing arrangements of the molecule;
(iii) ranking the generated crystal structures in order of likelihood of formation.

### 3.1. Methods of generating the molecular structure

There are two main approaches that can be used for treating the molecular structure during crystal structure prediction. Firstly, the molecule can be treated as rigid throughout the calculations, assuming that the packing forces are too small to significantly distort the molecular geometry. In this case the method used to determine the rigid molecular structure is vitally important, as the effect of the molecular structure on crystal energy calculations can be large (Beyer & Price, 2000 ▶).

Alternatively, the structure can be considered as flexible with intramolecular bond stretching, angle bending and torsional terms allowed to vary during the search as well as the final energy minimizations. For extremely flexible molecules such as target (XX) the conformational distributions can be reduced to a more manageable level *via* methods such as analysis of conformational preferences using software such as *Mogul* (Bruno *et al.*, 2004 ▶).

### 3.2. Generating trial crystal structures

There are many diverse methods for generating crystal packing arrangements in order to achieve a variety of plausible packing arrangements. Most participants in this blind test opted to generate large numbers of crystal structures with random or quasi-random variables such as unit-cell parameters and positions and orientations of the molecules. Several groups also elected to use a low-discrepancy Sobol’ sequence (Sobol’, 1967 ▶; Press *et al.*, 1992 ▶). This helps ensure a more uniform and thus efficient sampling and avoids the problems of gaps and clusters that purely random sampling can exhibit. Other groups used Monte Carlo types of search, genetic algorithms, grid-based systematic searches or first-principles *ab initio* random structure searching which allows the possibility of a change in covalent bonding (Pickard & Needs, 2006 ▶, 2011 ▶).

For the majority of these methods, space-group symmetry is used. These methods search each space group and *Z*′ separately and so in order to help reduce the computing time required, many groups chose to restrict their search to only the most commonly adopted space groups. This blind test saw two groups electing to search all 230 space groups for some or all of their predictions. Other groups used the alternative approach of generating *P*1 crystal structures with varying numbers of independent molecules (up to 8) in the unit cell. Space-group symmetry was then identified in the resulting crystal structures, after energy minimization, using packages such as *PLATON* (Spek, 2009 ▶).

### 3.3. Ranking of crystal structures

The final ranking of the crystal structures is still almost exclusively based on the calculated lattice energies of the structures generated by the crystal structure search. Often tens, if not hundreds, of possible structures can exist within a few kJ mol^−1^ of the calculated global minimum (Day *et al.*, 2004 ▶) and therefore extreme accuracy is needed. One successful approach to generating these lattice energies is the DFT-D method, which can give more accurate lattice energies (Neumann & Perrin, 2005 ▶) or re-minimization of the structures with more sophisticated force fields such as distributed multipoles (Stone, 2005 ▶) and additional flexibility (Kazantsev, Karamertzanis, Adjiman & Pantelides, 2011 ▶; Day & Cooper, 2010 ▶; Görbitz *et al.*, 2010 ▶). Moreover, additional or alternative criteria may be used to discriminate between likely and unlikely crystal structures. Such approaches include lattice dynamic contributions (van Eijck, 2001 ▶; Anghel *et al.*, 2002 ▶) or comparisons to known crystal structures in the CSD (Dey *et al.*, 2006 ▶), exploiting any isostructurality relationships (Asmadi *et al.*, 2010*a* ▶,*b* ▶).

## 4. Results

This paper is accompanied by a large amount of supplementary material: the coordinates of the experimental crystal structures, lists of predicted crystal structures by each participant, as well as detailed descriptions of methodology, results and post-analysis by most of the participating research groups. Before discussing the results of the predictions, the crystal packings in the X-ray determined crystal structures of the six categories are described.

### 4.1. Experimental crystal structures

#### 4.1.1. Molecule (XVI)

**Figure 1:**
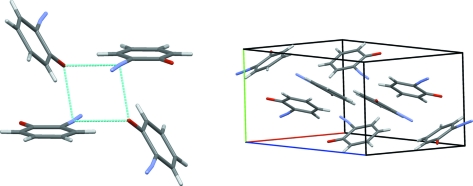
Packing diagram of the crystal structure of molecule (XVI). Grey = carbon, white = hydrogen, red = oxygen and blue = nitrogen. Contacts shorter than the sum of van der Waals radii are shown as blue lines.

2-Diazo-3,5-cyclohexadiene-1-one (C_6_H_4_N_2_O) was chosen as the blind test target for category 1 after the initial target, 4-ethynylbenzonitrile, was found to have been previously solved. Molecule (XVI) was crystallized by slow evaporation from ethanol and the crystal structure was solved from X-ray diffraction data collected at 174 K (Britton, 2010 ▶). The molecule crystallizes with *Z*′ = 1 in the orthorhombic space group *Pbca*. The crystal packing shows diazide-carbonyl and CH⋯O= interactions (Fig. 1 ▶).

#### 4.1.2. Molecule (XVII)

**Figure 2:**
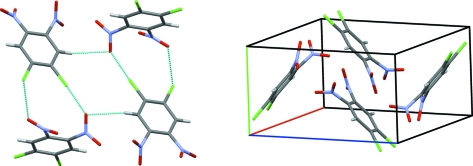
Packing diagram of the crystal structure of molecule (XVII). Grey = carbon, white = hydrogen, red = oxygen, blue = nitrogen and green = chlorine. Contacts shorter than the sum of van der Waals radii are shown as blue lines.

1,2-Dichloro-4,5-dinitrobenzene (C_6_H_2_Cl_2_N_2_O_4_) was chosen as the blind test target for category 2, although it deviates somewhat from the criteria for this category as the molecule is not truly rigid; the nitro groups allow for some degree of rotational freedom. Crystals were obtained by slow evaporation of methanol and X-ray diffraction data were collected at 174 K (Britton, 2010 ▶). The molecule crystallizes in the monoclinic space group *P*2_1_/*c* with *Z*′ = 1 (Fig. 2 ▶).

#### 4.1.3. Molecule (XVIII)

**Figure 3:**
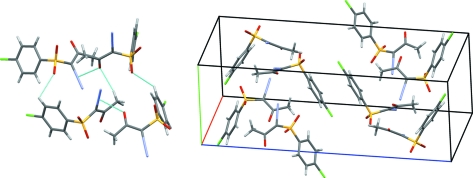
Packing diagram of the crystal structure of molecule (XVIII). Grey = carbon, white = hydrogen, red = oxygen, blue = nitrogen, green = chlorine and yellow = sulfur. Contacts shorter than the sum of van der Waals radii are shown as blue lines.

(1-((4-Chlorophenyl)sulfonyl)-2-oxo-propylidene)diazenium (C_9_H_7_ClN_2_O_3_S) was the target for category 3. Molecule (XVIII) was crystallized by slow evaporation from ethyl acetate (EtOAc) and the crystal structure was solved from X-ray diffraction data collected at 150 K (Blake, 2010 ▶). The crystal structure was solved in the orthorhombic space group *Pbca* with *Z*′ = 1. The conformational flexibility can be described by three exocyclic torsion angles, as shown in Table 1 ▶. The CN_2_CO moiety adopts a mostly planar *trans* configuration (Fig. 3 ▶).

#### 4.1.4. Molecular salt (XIX)

**Figure 4:**
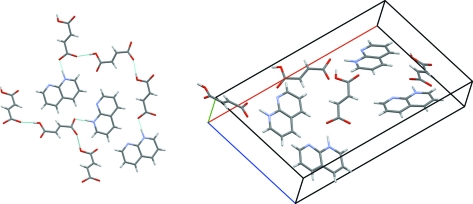
Packing diagram of the crystal structure of molecular salt (XIX). Grey = carbon, white = hydrogen, red = oxygen and blue = nitrogen. Hydrogen bonds are shown as blue lines.

1,8-Naphthyridinium fumarate (C_8_H_7_N_2_, C_4_H_3_O_4_) was chosen as the target for category 4. This 1:1 salt was formed by slow evaporation from methanol and the crystal structure was solved in the orthorhombic space group *Pca*2_1_ from data collected at 200 K (MacGillivray, 2010 ▶) with *Z*′ = 1. The packing in this crystal structure is dominated by hydrogen bonds, with linear chains of fumarate and naphthylpyridinium ions forming alternating connections to these chains (Fig. 4 ▶). The crystal structure is isostructural with the entry RABYID in the CSD (Shan *et al.*, 2003 ▶) where quinolinium is substituted for 1,8-naphthyridinium (*i.e.* one nitrogen is replaced by a C—H group).

#### 4.1.5. Molecule (XX)

**Figure 5:**
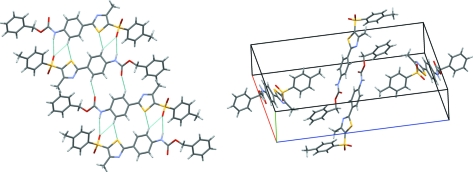
Packing diagram of the crystal structure of molecule (XX). Grey = carbon, white = hydrogen, red = oxygen, blue = nitrogen, green = chlorine and yellow = sulfur. Contacts shorter than the sum of van der Waals radii are shown as blue lines.

Benzyl-(4-(4-methyl-5-(*p*-tolylsulfonyl)-1,3-thiazol-2-yl)phenyl)carbamate (C_25_H_22_N_2_O_4_S_2_) was chosen as the target for the new category 5. Molecule (XX) was crystallized by slow evaporation from EtOAc and the crystal structure solved in the monoclinic space group *P*2_1_/*n* with *Z*′ = 1 (Blake, 2010 ▶). The conformational flexibility can be described with eight exocyclic torsion angles (Table 1 ▶). The molecule adopts an elongated S shape, with the central part of the molecule mostly planar, the greatest deviation from planarity being between the phenyl and thiazol groups with an angle of 13°. The mostly planar mid-section of the molecule forms stacks *via* a series of weak interactions with CH and NH⋯OS as well as CH⋯OC atom–atom contacts (*i.e.* shorter than the sum of van der Waals radii), as shown in Fig. 5 ▶.

#### 4.1.6. Polymorphic hydrate (XXI)

**Figure 6:**
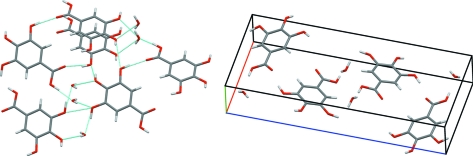
Packing diagram of the crystal structure of hydrate (XXI). Grey = carbon, white = hydrogen and red = oxygen. Hydrogen bonds are shown as blue lines.

Gallic acid monohydrate (C_7_H_6_O_5_·H_2_O) was chosen as the target for the new category 6. Gallic acid monohydrate had two previously known forms, (1) (Jiang *et al.*, 2000 ▶) and (2) (Okabe *et al.*, 2001 ▶). Form (4) of hydrate (XXI) was observed from crystals grown by slow evaporation from methanol in the presence of sarcosine and crystallized in the monoclinic space group *P*2_1_/*c* with *Z*′ = 1 (Clarke *et al.*, 2011 ▶). The crystal structure is dominated by an extensive hydrogen-bonding network. Unlike forms (1) and (3), no carboxylic acid dimer units are formed, with forms (2) and (4) instead having hydrogen bonds from the carboxylic acid to both water and adjacent gallic acid molecules (Fig. 6 ▶).

### 4.2. Comparison of the predictions with the experimental crystal structures

The submitted predictions were compared with each experimentally determined crystal structure using the ‘Crystal Structure Similarity’ feature of the Materials Module of *Mercury* (Macrae *et al.*, 2008 ▶). The algorithm used by this feature allows comparison of the molecular packing environment between two or more crystal structures. The reference crystal structure, in this case the experimentally determined crystal structure, is analysed and represented by a reference molecule and a coordination shell of its 14 closest neighbours. This set of distances is then searched for in the predicted crystal structures and if they match to within the default geometric tolerances (distances within 20% and angles within 20°) then the coordination shells are overlaid and a root-mean-squared deviation (RMSD_15_) of the atomic positions is calculated for all matching molecules. As with previous blind tests, this search was configured to ignore H atoms due to the uncertainty of their positions in X-ray determined crystal structures. If all 15 molecules of the reference and predicted crystal structure matched within the standard tolerances, the crystal structure was determined as having been successfully predicted.

For hydrate (XXI) it became apparent that some predictions matched all non-H atoms but not the H-atom positions as located in the target crystal structure. For this molecule we therefore re-ran the crystal structure comparison, but this time elected to include H atoms in the calculation in order to determine if an exact match was present.

Overlays of the X-ray determined crystal structure with some of the predicted structures for targets (XVI)–(XX) can be found in the supplementary material.

### 4.3. Predictions results

#### 4.3.1. Molecule (XVI)

All of the participating research groups attempted predictions for molecule (XVI), two of whom predicted the observed crystal structure within their three predictions (Table 3 ▶). One of these successes (Neumann, Leusen, Kendrick and van de Streek) was submitted as the group’s first prediction, while the other (van Eijck) was submitted as the participant’s second prediction. Both of these successful predictions gave RMSD_15_ deviations from the experimentally determined crystal structure of less than 0.25 Å.

Outside of the three official predictions, the observed crystal structure was present in the extended lists of five other research groups. The success rates here are comparable to the first three blind tests, while not quite as high as the results observed in the fourth blind test. This may be attributed to some methods having difficulties with many structures close in energy. The very small Δ*E* in Table 3 ▶, even when the observed structure is found outside of the first three predictions, shows how closely spaced the energies are for this molecule, and the accuracy in lattice energy required for a successful prediction.

#### 4.3.2. Molecule (XVII)

13 of the participating research groups attempted predictions for molecule (XVII), two of which predicted the observed crystal structure within their three official predictions (Table 4 ▶). As with molecule (XVI), one of these successes (Neumann, Leusen, Kendrick and van de Streek) was submitted as the group’s first prediction, while the other (Price and Habgood) was submitted as that group’s second prediction. Both of these successful predictions gave RMSD_15_ values of less than 0.13 Å.

Four other research groups submitted the observed crystal structure in their extended list of solutions, with energies between 3.2 and 6.4 kJ mol^−1^ above their global minimum. The slightly lower rate of success for this category than for the last blind test may be attributed to the fact that molecule (XVII) is not truly rigid, with flexibility in the nitro groups having to be taken into consideration. Despite these additional challenges, the observed crystal structure was still successfully predicted.

#### 4.3.3. Molecule (XVIII)

13 research groups attempted predictions for the category 3 target, molecule (XVIII), with one group (Neumann, Leusen, Kendrick and van de Streek) successfully predicting the observed crystal structure within their three predictions (Table 5 ▶). Once again, this solution was submitted as this group’s first submission, with an RMSD_15_ from the observed crystal structure of just 0.12 Å.

Three other groups also reported the correct crystal structure in their extended lists of solutions, with one group (Orendt, Grillo, Ferraro and Facelli) close to having a successful prediction as their number 4 structure is a close match to the experimental structure with an RMSD_15_ value of 0.252 Å.

#### 4.3.4. Molecular salt (XIX)

11 participants attempted predictions for the molecular salt (XIX) and two of these predicted the observed crystal structure within the three official predictions (Table 6 ▶): van Eijck as the second prediction and Neumann, Leusen, Kendrick and van de Streek as the third prediction, with RMSD_15_ values of 0.15 and 0.22 Å. Two other participants located the crystal structure within their extended lists of submissions.

The rate of success in searching for structures with two independent molecules in the asymmetric unit is broadly comparable with that of the last blind test. However, the energetic ranking of the salt structures provided a greater challenge than was experienced with the cocrystal used in 2007. The most successful prediction relied on the use of a supramolecular dimer owing to difficulties with modelling individual ions. Comparison with predictions and the known crystal structure of the similar compound present in CSD entry RABYID also helped to weight some predictions, including the third placed submission made by Neumann, Leusen, Kendrick and van de Streek, which would have been ranked at position 20 by energy alone.

#### 4.3.5. Molecule (XX)

Ten participants attempted predictions for molecule (XX) and two of these predicted the observed crystal structure as their top submission (Day and Cruz-Cabeza; Price, Kazantsev, Karamertzanis, Adjiman and Pantelides). One other group (Neumann, Leusen, Kendrick and van de Streek) also located the observed crystal structure in its extended list of solutions (Table 7 ▶) at rank 7.

This category was introduced in this blind test as a new challenge and so there are no results from any previous blind tests with which to compare. However, this does appear to be the first case of a molecule of this complexity having been successfully predicted under blind test conditions and then detailed in a refereed publication. The key dependence was on the conformation of the molecule and with eight internal degrees of freedom the problem became one of completeness of the search. One team resolved this by taking into account CSD observations for each of the flexible components to reduce the search to a more manageable size.

#### 4.3.6. Polymorphic hydrate (XXI)

**Figure 7:**
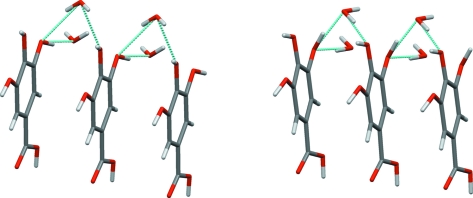
Alternative hydrogen-bond networks possible in (XXI). The left image shows the hydrogen bonds as defined in the crystal structure [form (4_expt_)], the right image shows the alternative network as located by some participants [form (4_alt_)]. Hydrogen bonds are shown as blue lines.

Ten participants attempted predictions for the hydrate (XXI). This category featured the opportunity to find and locate both an unknown polymorph and two polymorphs whose crystal structures had previously been determined. During analysis of the results it became apparent that there is an alternative proton arrangement in the hydrogen-bonding network of form (4) involving the central OH moiety of the acid and the water molecules (see Fig. 7 ▶). Solutions with both proton conformations were generated by some groups, but no agreement was observed in which form had the lower energy.

**Figure 8:**
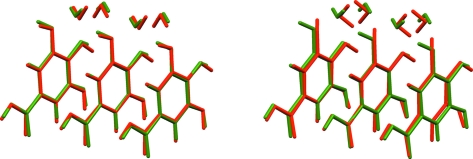
Overlay of the unit-cell contents of the observed crystal structure (XXI) (green) and Day *et al.* (XXI).12 (red, left image). RMSD 0.159 Å, and van Eijck (XXI).1 (red, right image), RMSD 0.219 Å

In previous blind tests, H-atom placement has been ignored in determining if a participant’s entry matches the target crystal structure, but in this case it was evident that the two groups that submitted a match within their top three submissions (Price and Braun; van Eijck) did so with the *p*-hydroxy conformation of form (4)_alt_, not that of the target crystal structure form (4)_expt_ (Fig. 8 ▶). As the *p*-hydroxy gallic acid proton shows enlarged displacement parameters, it could be argued that some disorder is present in the structure.

Given this, we present here results for both exact matches including H-atom placement (Table 8 ▶ *a*) and matches for non-H atoms only (Table 8 ▶ *b*). No groups submitted an exact match in their top three solutions. Four groups (Day; van Eijck; Neumann *et al.*; Price and Braun) had exact matches within their extended lists of submissions. For matches involving only the non-H atoms, two groups located the target crystal structure within their top three solutions (van Eijck; Price and Braun) as their first and third submissions respectively. Both of these groups also located the exact match, but at significantly higher energies of approximately 12 kJ mol^−1^ above their global minimum. Three other groups (Desiraju *et al.*; Day; Neumann *et al.*) also located this crystal structure in their extended lists of submissions.

Tables 8 ▶(*c*) and (*d*) show successful matches for the existing polymorphs [forms (1) and (2) in this test]. Six groups located form (1) in their extended lists of submissions, and five groups located form (2). These were generally predicted at high relative energies and rankings, and with no consistency between groups on the stability order between form (1) and (2). This highlights problems in modelling the stability of hydrates.

### 4.4. Computational expense

Table 9 ▶ summarizes the approximate computational resources used by some of the participants. Of particular note is the disparity between some of the groups; the range of computational expense seen in CSP2010 varies from a few thousand CPU hours to almost 200 000 CPU hours (which translates to over 22 CPU years). Clearly the resources required for this blind test have increased. A large portion of the total CPU time was devoted to targets (XX) and (XXI), and is therefore clearly dependent upon the complexity of the molecule. Fortunately, the computer systems required to meet this increased need are also now more readily accessible, as shown by several groups reporting increases of computing resource of over an order of magnitude (and sometimes almost two orders of magnitude) over the resources used for their CSP2007 submissions. As computers get progressively faster and with greater numbers of computing cores per processor, the real time required for these computations is decreasing. This makes modern computers more viable for fast prediction of the simpler targets.

## 5. Discussion

### 5.1. Overall success rates

The success rate for previous blind tests has shown a fluctuating, but generally upward trend, with particular success shown in the fourth blind test (Day *et al.*, 2009 ▶). This fifth blind test was designed to see if the successes of the fourth test could be repeated, and also to provide more challenging targets to try to stretch the techniques that have thus far been developed. This test therefore saw the introduction of more flexible molecules, as well as hydrates and salts, significantly increasing the complexity of the challenge.

Success for these tests is a combination of two factors: Firstly the ability to generate all possible crystal structures, and secondly the ability to evaluate and rank those crystal structures. The search performance can be impacted by methods that are presently unable to search for crystal structures in space groups with higher values of *Z*, or simply through a lack of computing time and resources. This will lead to an incomplete search space, which may cause the correct solution to be missed entirely. For flexible molecules the conformation of the molecule is also of great importance. Failure to use the correct conformation or to allow for flexibility during the search will lead to failure to predict the correct crystal structure, and this problem becomes greater the more flexible the target molecule. Lastly, the crystal structures generated must be ranked, which is often complicated by the fact that most molecules tend to have many distinct crystal packing possibilities within a small energy range (Day *et al.*, 2004 ▶), so that the energy differences between crystal structures are generally very small. The identification and use of accurate energy models can often prove to be the most challenging aspect of successful crystal structure prediction. Ranking is further complicated by thermodynamic kinetic aspects, *i.e.* energies alone may not be sufficient; entropies and nucleation kinetics could also be relevant.

Of the groups that participated in the fifth blind test, most attempted solutions for the four targets [(XVI), (XVII), (XVIII) and (XIX)] that matched the criteria of the previous blind test. Overall, the success rates for these four targets were a little lower than for CSP2007, but generally at least as good if not better than the results obtained for CSP1999, CSP2001 and CSP2004. What these results do show, however, is that just as in CSP2007, the method adopted by Neumann, Leusen, Kendrick and van de Streek again excelled, with this group able to successfully predict the crystal structures of the first three categories with their number 1 submission, as well as the fourth category with their number 3 submission. They were the only participants able to generate all target crystal structures within their extended list of submissions. They did so with the lowest RMSD_15_ values for all except the hydrate crystal structure. This demonstrates the reliability of DFT-D methods to predict the crystal structures of small organic molecules (Asmadi *et al.*, 2009 ▶; Chan *et al.*, 2011 ▶). For the fourth category, complete crystal-structure prediction studies were performed for (XIX) and for model compound RABYID from the CSD. The energy landscapes of these two systems were analysed and showed significant similarities. Based on these similarities, it had to be concluded that the experimental structure of (XIX) could be isostructural to the experimental structure of RABYID, and this structure, even though it was ranked 20th by energy (22nd for RABYID), was submitted as the third candidate structure (Kendrick *et al.*, 2011 ▶).

More complex systems such as salts continue to provide some challenge, perhaps suggesting that a salt should be considered a new, more challenging category than the current ‘cocrystal’ definition of category four.

This test also introduced two new categories that provided much greater challenges to the participants and 11 out of the participating groups attempted at least one of the targets (XX) and (XXI). Particularly encouraging was that two groups (Price *et al.*; Day *et al.*) successfully predicted the crystal structure for the largest, most flexible molecule to be included in this series of blind tests. The hydrate target (XXI) proved to be a considerable challenge, even to methods that have been successful for hydrates of *o*-dihydroxybenzoic acids (Braun *et al.*, 2011 ▶), and highlights the many difficulties that such a system can pose to characterization as well as successful structure prediction. However, this is a system that needs to be tackled; water is one of the most complex solvents to model, yet it is also one of the most important.

This blind test has also once again highlighted that the use of generic standard force fields does not lead to good crystal structure prediction results. We have also observed that the more extensive search methods are adequate within the limitations (*Z*′, no disorder *etc.*) implicit in the blind test categories, but have to assume the covalent bonding in the chemical diagram and rely on a sufficient number of search structures being refined by the more accurate and expensive model for the lattice energy. Successful prediction of small molecule crystal structures has been shown to require both accuracy of energies and the ability to coordinate the inter- and intramolecular force field contributions. The methods that gave the greatest success were varied and were modified to take on these tougher challenges.

### 5.2. Challenges faced

Molecule (XVI), while the simplest of the rigid molecule targets, proved to have many crystal structures close in energy. Transferable empirical potentials had difficulty coping with diazide–carbonyl interactions with induction being a problem. Simple point-charge models used by some groups failed completely for this molecule, although van Eijck did find in post-analysis that one set of charges predicted the observed structure.

This system is the first to be tackled by an *ab initio* random search method (Misquitta, Pickard and Needs) which does not fix the chemical bonding and uses electronic structure methods during the search, although this approach results in a significant increase in the computing resources required when compared with the methods employed by the other participating groups. This method failed as the search was not extended to eight formula units in the cell. Many of the numerous minima, including the global minimum, corresponded to an isomer with the formation of a bond to give a heterocyclic ring with the two N atoms, showing the promise of this method for cases, such as tautomers, where the covalent bonding is uncertain.

Molecule (XVII) was perhaps not well selected as a target for its category as the molecule was not truly rigid; the orientation of the nitro groups may be affected by intermolecular interactions in the crystal structure. As a result participants were forced to first consider how to deal with this flexibility. The electrostatic potentials of the nitro groups also proved to be unusually challenging to model successfully, although the dispersion proved to be a very important contribution to the lattice energy. These additional issues lead to molecule (XVII) being a significantly more difficult problem than previous targets in this category. Despite these extra challenges, the success rate for this category was good compared with previous blind tests.

For molecule (XVIII) flexibility proved to be the key to successfully locating the crystal structure in the search. Some searches missed the crystal structure, with the most fundamental reason being the wrong conformation of the C(N_2_)C(O) bond. The relative energies of the *cis* and *trans* configurations were sufficiently sensitive to the methods being used to cause mis-assignment.

For the salt (XIX), there were again some difficulties with flexibility with the relative orientation of the two fragments; for the acid there is considerable conformational flexibility and the calculated stability of the conformers alters between the gas and solid.

All groups encountered significant problems with developing suitable methods of evaluating the relative lattice energies of structures containing the different conformers. Plane-wave *ab initio* methods do not cope well with isolated ions in a vacuum, causing problems with ion-specific reference data calculated with DFT-D methods for force-field parameterization. Induction and charge transfer, which are stronger in molecular salts, limited the transferability of *exp*-6 potentials which had been fitted to crystal structures of neutral molecules. Successful prediction based on energy (van Eijck) was achieved by the use of a supramolecular dimer, rather than two ions as individual molecules.

Other groups noted the similarity of an existing crystal structure in the CSD, RABYID, the crystal structure of which consisted of the same fumarate ion but with a quinolinium counterion instead of 1,8-naphthyridinium (*i.e.* where the unprotonated nitrogen is instead a CH moiety). The energy landscapes generated for both salts proved similar enough to encourage the speculation that the two crystal structures could be isostructural and one group (Neumann *et al.*) submitted a successful prediction based on this approach (Kendrick *et al.*, 2011 ▶).

Molecule (XX) was the first large flexible molecule to feature in the blind tests and proved a considerable challenge. For such highly flexible molecules there is a key dependence on the conformation of the molecule and successful prediction involved succeeding at this early step. One of the main difficulties is the computing power required to make a complete search for all available space groups with a flexible molecule; when all standard orientations about the exocyclic single bonds are considered, there are over one thousand possible conformations. The two successful strategies (Day and Cruz-Cabeza; Price, Kazantsev, Karamertzanis, Adjiman and Pantelides) reduced the search space to a more manageable level, producing innovations in methodology that have been described and contrasted in detail elsewhere (Kazantsev, Karamertzanis, Adjiman, Pantelides, Price, Galek, Day & Cruz-Cabeza, 2011 ▶). Day *et al.* used geometry data for similar systems from the CSD to help limit the search further and considered a set of predefined conformations. Price *et al.* identified likely ranges of values for the flexible torsions and used an extension to the *CrystalPredictor* methodology and databases of the *ab initio* calculations on the isolated molecule to allow the crystal structures and conformations to be simultaneously refined (Kazantsev, Karamertzanis, Adjiman & Pantelides, 2011 ▶). Neumann *et al.* employed a fully flexible molecule, allowing all conformations to be explored during the crystal structure generation step. Use of multipoles and empirical potentials performed better than DFT-D in this case, with both groups using this method (Day *et al.*; Price *et al.*) successfully predicting the crystal structure in first place.

Hydrate (XXI) proved to be one of the most challenging systems in the blind test. For this molecule two known polymorphs already existed. However, the difficulty in predicting this crystal structure was not due to the availability of two already known polymorphs, but rather that the representation of water–water and water–gallic acid interactions is extremely difficult to model, making the successful prediction of even the known polymorphs a difficult task.

As a hydrate, the hydrogen-bonding network enabled by the water molecules and the various hydrogen-bond donors and acceptors in the acid proved key to successfully predicting the crystal structure, but it is also obvious that the sheer number of different possible hydrogen-bond networks make the problem a difficult one. The results obtained for form (4) show that with the same placement of non-H atoms there is more than one set of hydrogen positions that is possible. Energetically, the OH conformation observed in the experimental structure is not the most favourable in isolation and, given the nature of X-ray diffraction, the positions of these protons cannot be deemed as unequivocally determined. Indeed, there is evidence of large displacement parameters for the protons involved in the two alternative hydrogen-bond networks. This leads us to consider that the structure is best described as disordered with respect to which network is present. This matter would only be resolved with an in-depth temperature-dependent X-ray and NMR study. A post-blind test polymorphism screen (Braun, Personal communication) showed that the ordered form (2) structure is the most stable polymorph at room temperature.

Overall, the systems that gave the most difficulty are those where the molecules can adopt very different low-energy conformations, where current methods may not accurately reflect the energy differences between the conformations in the solid state. Work on improving the estimates of polymorphic energy differences in challenging cases where the polymorphs have different numbers of inter- and intramolecular hydrogen bonds (Karamertzanis *et al.*, 2008 ▶) shows that improving the theoretical basis of the methods used to evaluate the lattice energies will lead to further progress.

## 6. Conclusion

This fifth blind test has built upon the successes of previous blind tests and shows that a state-of-the-art method for crystal structure prediction is able to reliably predict crystal structures of small rigid and slightly flexible molecules, and methods are emerging that are able to tackle larger more flexible molecules and complex systems such as salts and hydrates.

For each of the six target crystal structures, there was at least one successful prediction under the criteria stated for success at the start of the test [although for the hydrate (XXI) certain protons were incorrectly placed]. The number of successful predictions for each of the first four categories was broadly comparable with the first three blind tests, but slightly less than the great successes observed with CSP2007. This may be due to the difficulty of easily gauging a target’s difficulty based on its molecular diagram alone – several of the targets in this blind test showed additional challenges not faced in CSP2007 even though the target molecule met the same selection criteria. One observation that is easily made, however, is that the DFT-D method continues to perform very well for these molecule types, although it does not yet supply a comprehensive solution, as observed by the inability to predict some targets [such as (XIX)] by energy methods alone. Most other successes were based on using realistic models for the intermolecular forces (Stone, 1996 ▶), which included a distributed multipole representation of the molecular charge distribution.

For the large flexible molecule [target (XX)] it is promising that two groups were able to successfully predict the crystal structure as their first place entry. In both cases success was achieved by systematic reduction of the problem to more manageable proportions, such as through the use of CSD geometry data to determine the more likely conformations in the experimental crystal structure. More methodological and program development should allow the most thermodynamically stable crystal structures to be computed more readily for molecules of this complexity in the future. The best approach for such complex systems may well be the use of experimental data, including polymorph screening, alongside the calculations to move towards a predictive technology for the understanding and anticipation of polymorphism.

The difficulties faced in this blind test have helped push the participating teams to adopt novel approaches in an attempt to successfully predict the experimental crystal structures. While some challenges remain, such as the need to include a direct consideration of temperature (thermodynamics prescribes that relative stability is a function of temperature), the results achieved in this blind test demonstrate that crystal structure prediction can now be performed reliably for small molecules using a state-of-the-art method. Furthermore, results on the large molecule [target (XX)] as well as the salt [target (XIX)] and the hydrate [target (XXI)] provide encouragement that crystal structure prediction can move on from prediction of small rigid molecules to more complex systems, while highlighting deficiencies in current methods where key developments are still required.

## Supplementary Material

Supplementary material file. DOI: 10.1107/S0108768111042868/bk5106sup1.txt

Supplementary material file. DOI: 10.1107/S0108768111042868/bk5106sup2.zip

Supplementary material file. DOI: 10.1107/S0108768111042868/bk5106sup3.zip

Supplementary material file. DOI: 10.1107/S0108768111042868/bk5106sup4.pdf

## Figures and Tables

**Table 1:** Diagrams and crystallization conditions for the targets of CSP2010

Target		Crystallization conditions
(XVI)	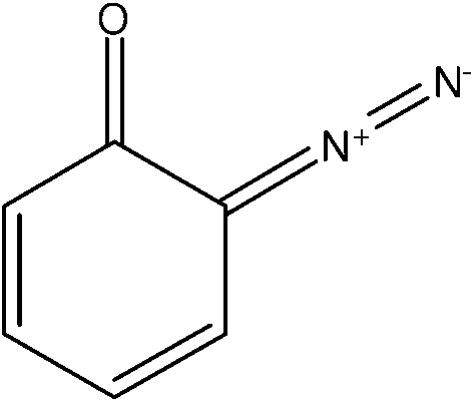	Slow evaporation from ethanol
	2-Diazo-3,5-cyclohexadiene-1-one	
(XVII)	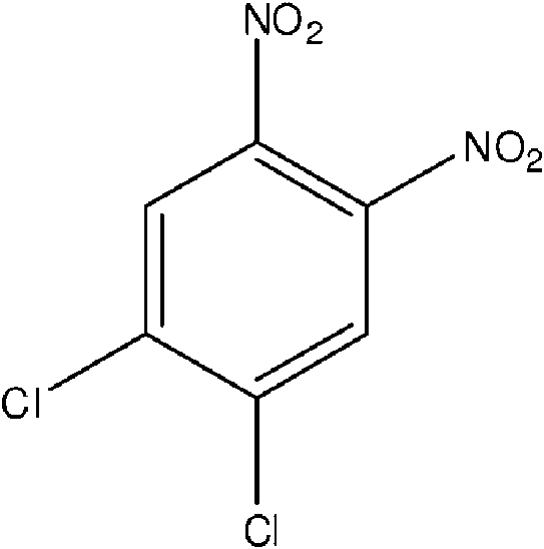	Slow evaporation from ethanol/acetone
	1,2-Dichloro-4,5-dinitrobenzene	
(XVIII)	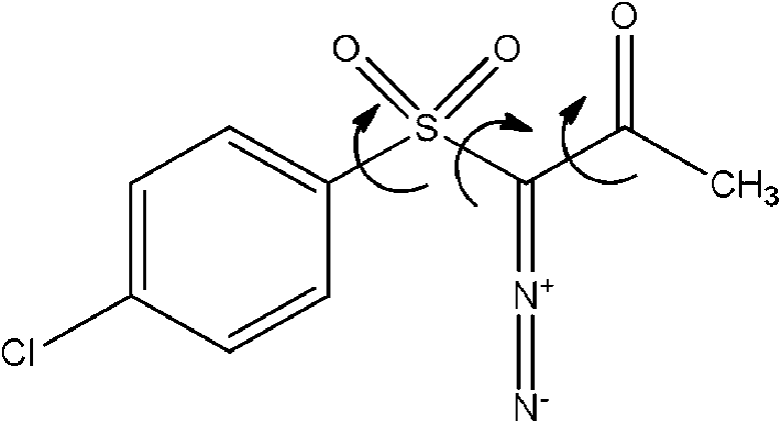	Slow evaporation from EtOAc
	(1-((4-Chlorophenyl)sulfonyl)-2-oxopropylidene)diazenium	
(XIX)	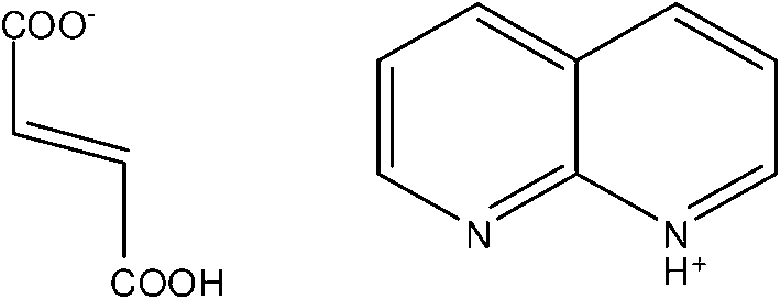	Slow evaporation from methanol
	1,8-Naphthyridinium fumarate	
(XX)	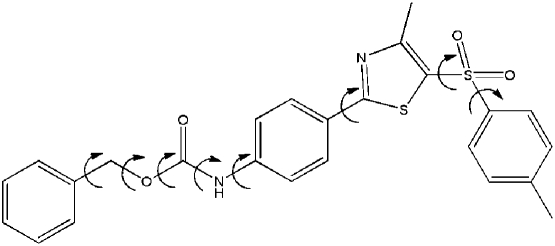	Slow evaporation from EtOAc
	Benzyl-(4-(4-methyl-5-(*p*-tolylsulfonyl)-1,3-thiazol-2-yl)phenyl)carbamate	
(XXI)	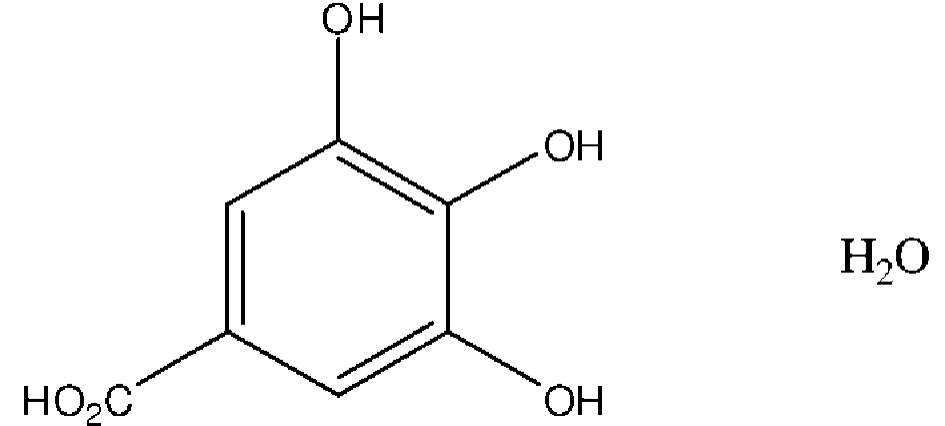	Form (3): Slow evaporation from water
	Gallic acid monohydrate	Form (4): Slow evaporation from methanol in the presence of sarcosine

**Table:** Entries with a name and no number denote an attempt at prediction without success, a dash indicates no attempt at prediction.

Group	(XVI)	(XVII)	(XVIII)	(XIX)	(XX)	(XXI)†
1	Boerrigter 302	Boerrigter 121	Boerrigter	Boerrigter 12	Boerrigter	Boerrigter
2	Day 8	Day 4	Day	Day, Cruz-Cabeza 27	Day, Cruz-Cabeza 1	Day 12 (61)
3	Della Valle, Venuti	Della Valle, Venuti	Della Valle, Venuti	Della Valle, Venuti	Della Valle, Venuti	Della Valle, Venuti
4	Desiraju, Thakur, Tiwari, Pal	Desiraju, Thakur, Tiwari, Pal 65	Desiraju, Thakur, Tiwari, Pal 13	Desiraju, Thakur, Tiwari, Pal	–	Desiraju, Thakur, Tiwari, Pal (11)
5	van Eijck 2	van Eijck 6	van Eijck	van Eijck 2	van Eijck	van Eijck 29 (1)
6	Facelli, Grillo, Ferraro, Orendt 9	Facelli, Grillo, Ferraro, Orendt	Facelli, Grillo, Ferraro, Orendt 4	Facelli, Grillo, Ferraro, Orendt	Facelli, Grillo, Ferraro, Orendt	Facelli, Grillo, Ferraro, Orendt
7	Hofmann, Hofmann, Kuleshova	Hofmann, Hofmann, Kuleshova	Hofmann, Hofmann, Kuleshova	Hofmann, Hofmann, Kuleshova	Hofmann, Hofmann, Kuleshova	Hofmann, Hofmann, Kuleshova
8	Jose	Jose	Jose	–	–	–
9	Maleev, Zhitkov	Maleev, Zhitkov	Maleev, Zhitkov	Maleev, Zhitkov	Maleev, Zhitkov	Maleev, Zhitkov
10	Misquitta, Pickard, Needs	–	–	–	–	–
11	Neumann, Leusen, Kendrick, van de Streek 1	Neumann, Leusen, Kendrick, van de Streek 1	Neumann, Leusen, Kendrick, van de Streek 1	Neumann, Leusen, Kendrick, van de Streek 3	Neumann, Leusen, Kendrick, van de Streek 7	Neumann, Leusen, Kendrick, van de Streek 89 (174)
12	Nikylov, Bartashevich	–	–	–	–	–
13	Price, Misquitta 15	Price, Habgood 2	Price, Price	Price, Mohamed	Price, Kazantsev, Karamertzanis, Adjiman, Pantelides 1	Price, Braun 117 (3)
14	Scheraga, Arnautova	Scheraga, Arnautova	Scheraga, Arnautova 29	Scheraga, Arnautova	–	–

**(b):** Summary of methodologies

						Lattice energy / fitness function	
Group	Programs	Refs	Search generation	Space groups considered	Molecular model	electrostatic	other
1	*Materials Studio* 5.0	(*a*)	Monte Carlo simulated annealing	(XVI)–(XIX), (XXI): 88 most common groups (XX): 49 most common space groups with 1 molecule and 33 most common space groups with 2 independent molecules	Flexible throughout	Atomic charges fitted to the electrostatic potential obtained from the DMol3 geometry optimized molecular model (DFT method: GGA-BLYP, double numerical basis set with polarization)‡	Dreiding 2.21
2	*Crystal Predictor*, *Cerius*2, *DMAREL*, *DMACRYS*	(*b*)	Structures generated using a low discrepancy Sobol’ sequence	*P*1, 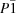 , *P*2_1_, *P*2_1_/*c*, *P*2_1_2_1_2_1_, *P*2_1_2_1_2, *P*2_1_/*m*, *P*2/*c*, *P*2_1_3, *P*4_1_, *P*4/*n*, *P*4_2_/*n*, *P*4_1_2_1_2, 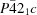 , *P*3_1_, *P*3_1_21, 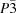 , *P*6_1_, *P*6_3_/*m*, *Pna*2_1_, *Pca*2_1_, *Pbca*, *Pbcn*, *Pmn*2_1_, *Pnna*, *Pccn*, *Pbcm*, *Pnnm*, *Pmmn*, *Pnma*, *Pc*, *Pa*3, *C*2/*c*, *Cc*, *C*2, *Cm*, *C*2/*m*, *C*222_1_, *Cmc*2_1_, *Cmcm*, *Cmca*, 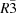 , *R*3, *R*3*c*, 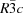 , *Aba*2, *Fdd*2, *Fddd*, *Iba*2, *Ibam*, 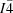 , *I*4/*m*, *I*4_1_/*a*, 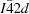	(XVI) Rigid throughout, (XVII)–(XXI) partly flexible	Atomic multipoles	Specifically fitted anisotropic *exp*-6
3	*Xfind*, *WMIN*, *GULP*, *PLATON*, *SIESTA*	(*c*)	Structures generated using a low discrepancy Sobol’ sequence	(XVI): All 230 space groups (XVII)–(XXI): *P*1, 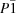 , *P*2_1_, *P*2_1_/*c*, *C*2/*c*, *P*2_1_2_1_2_1_, *Pna*2_1_, *Pbca*, *Pnma*	Rigid for search, flexible for energy minimization	Atomic charges	Empirical *exp*-6
4	*Materials Studio* 5.0	(*d*)	Monte Carlo simulated annealing	*P*1, 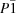 , *P*2_1_, *C*2, *Cc*, *P*2_1_/*c*, *C*2/*c*, *P*2_1_2_1_2_1_, *Pca*2_1_, *Pna*2_1_, *Pbcn*, *Pbca*, *Pnma*	Rigid for search, flexible for energy minimization	Atomic charges	Dreiding *exp*-6 [(XVI), (XVIII)] COMPASS force field [(XVII), (XIX), (XXI)]
5	*UPACK*, *XTINKER*, *GAMESS-UK*, *MOLDEN*	(*e*)	Randomly generated starting structures	*P*1, *P*2_1_/*c*, 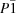 , *P*2_1_, *P*2_1_2_1_2_1_, *C*2/*c*, *Pbca*, *Pbcn*, *Pna*2_1_, *Pca*2_1_, *Cc*, *C*2, *Pc*	Flexible throughout	Atomic charges [(XIX), (XX)] atomic multipoles (others)	Empirical *exp-6* (inter), *ab*-*initio* energies (intra)
6	*MGAC*	(*f*)	Modified genetic algorithm	*P*1, 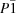 , *P*2_1_, *C*2, *Pc*, *Cc*, *P*2_1_/*c*, *C*2/*c*, *P*2_1_2_1_2_1_, *Pca*2_1_, *Pna*2_1_, *Pbcn*, *Pbca*, *Pnma*	Flexible throughout	Atomic charges	GAFF *6–12*
7	*FlexCryst*	(*g*)	Random search with calibrated cell	*P*2_1_/*c*, 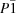 , *C*2/*c*, *P*2_1_2_1_2_1_, *P*2_1_	Rigid throughout	Trained potentials	
8	*GA*-*CG*-*MTA*	(*h*)	Genetic algorithm	*P*1, 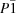 , *P*2_1_, *C*2, *Pc*, *Cc*, *P*2_1_/*c*, *P*2_1_2_1_2_1_, *Pca*2_1_, *Pna*2_1_, *Pbcn*, *Pbca*, *Pnma*	Flexible throughout	EPIC	CG-MTA
9	*DMM*	(*i*)	Discrete modeling method of molecular packings	All space groups with *Z* ≤ 4	Rigid for search and for energy minimization	Empirical *exp*-6	
10	*CASTEP*	(*j*)	*Ab initio* random structure searching	All space groups with *Z* ≤ 4	Rigid in searches using potential, flexible in DFT-D optimizations and AIRSS searches	Plane-wave density functional theory supplemented with an empirical dispersion correction	
11	*GRACE* 1.5–1.6 and *VASP*	(*k*)	Monte Carlo parallel tempering	All 230 space groups	Flexible throughout	Plane-wave density functional theory supplemented by an empirical C_6_*R*^−6^	
12	*OPIX*	(*l*)	Random search without molecular flexibility	*P*2_1_/*c*, 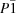 , *P*2_1_, *P*2_1_2_1_2_1_, *C*2/*c*, *Pbca*	Rigid throughout	Empirical exp-6 without atomic charges§	
13	*Crystal Predictor*, *DMACRYS*, *CrystalOptimizer*	(*m*)	Sobol sequences, flexible or rigid	(XVI)–(XIX): 59 most common groups; (XX): 12 most common groups; (XXI): 24 most common groups	(XVI) Rigid, (XVII)–(XXI) Flexible	Atomic multipoles	Empirical *exp*-6 (XVII)–(XXI) non-empirically derived anisotropic *exp*-6 (XVI)¶
14	*CRYSTALG*, *PLATON*	(*n*)	Conformation-Family Monte Carlo	No symmetry information used – *P*1 with varying *Z* (= 2, 4, 8)	(XVI)–(XIX) Rigid throughout	Atomic charges	ECEPP-05 [(XVI), (XVIII), (XIX)] Empirical *exp*-6 (XVII)

References: (*a*) Verwer & Leusen (1998 ▶), Karfunkel *et al.* (1996 ▶); (*b*) Karamertzanis & Pantelides (2007 ▶), Price *et al.* (2010 ▶), Day *et al.* (2007 ▶), Cooper *et al.* (2008 ▶); (*c*) Busing & Matsui (1984 ▶), Gale & Rohl (2003 ▶), Soler *et al.* (2002 ▶); (*d*) Sarma & Desiraju (2002 ▶), Dey *et al.* (2005 ▶, 2006 ▶); (*e*) Mooij *et al.* (2000 ▶), van Eijck & Kroon (2000 ▶), van Eijck *et al.* (2001 ▶), van Eijck (2002 ▶); (*f*) Bazterra *et al.* (2007 ▶); (*g*) Hofmann & Lengauer (1997 ▶), Hofmann & Apostolakis (2003 ▶), Hofmann & Kuleshova (2005 ▶); (*h*) unpublished method – see supplementary material; (*i*) Maleev (1995 ▶, 2001 ▶), Maleev *et al.* (2005 ▶, 2009 ▶); (*j*) Clark *et al.* (2005 ▶), Pickard & Needs (2006 ▶), Pickard & Needs (2011 ▶); (*k*) Neumann & Perrin (2005 ▶), Neumann (2008 ▶, 2011 ▶), Kresse & Furthmüller (1996 ▶), Kresse & Hafner (1993 ▶), Kresse & Joubert (1999 ▶); (*l*) Gavezzotti (2003 ▶), Gavezzotti & Filippini (1997 ▶), Gavezzotti (2002 ▶); (*m*) Karamertzanis & Pantelides (2007 ▶), Price *et al.* (2010 ▶), Kazantsev, Karamertzanis, Adjiman & Pantelides (2011 ▶), Kazantsev *et al.* (2010 ▶); (*n*) Pillardy *et al.* (2001 ▶).

† The numbers in parentheses denote matches ignoring H-atom placement, all other values denote exact matches.

‡ For (XIX) the anion–cation cluster was used as the molecular model for the DFT calculations and subsequent ESP charges. The molecules were treated independently during the sampling.

§ Application of topological characteristics of electron density, calculated for generated structures and comparison with the structures from the CCDC with the same functional groups

¶ Choices 2 and 3 considered properties and motif including free energy.

**Table 3:** Lattice parameter deviations (predicted − experimental), Δ*E* and RMSD for the experimental and predicted structures of molecule (XVI) α = β = γ = 90° in all structures.

	Rank	Δ*E*† (kJ mol^−1^)	Density (g cm^−3^)	*a* (Å)	*b* (Å)	*c* (Å)	RMSD_15_‡ (Å)
Expt. (*T* = 174 K)	–	–	1.385	9.645 (2)	7.381 (1)	16.185 (3)	–
							
Predicted amongst first three							
Neumann, Leusen, Kendrick, van de Streek	1	−0.70§	−0.9%	−1.6%	+1.7%	+0.7%	0.157
van Eijck	2	+0.06	−3.7%	+5.3%	−0.6%	−0.8%	0.247
							
Present in list, outside of first three predictions							
Day	8	+1.16	−4.8%	+6.4%	−1.2%	–0.1%	0.273
Orendt, Grillo, Ferraro, Facelli	9	+2.45	−2.1%	+6.6%	−3.2%	−1.1%	0.306
Price, Misquitta	15	+5.74	–4.9%	+14.5%	−7.1%	−1.1%	0.633
Boerrigter	302	+3.38	−5.3%	+4.7%	+0.5%	+0.3%	0.190

† Δ*E* is calculated with respect to the lowest energy structure predicted by the same research group.

‡ RMSD_15_ is calculated using a 15 molecule comparison in the Materials Module of *Mercury*, ignoring H atoms.

§ Δ*E* for the global minimum is calculated with respect to the second lowest energy structure.

**Table 4:** Lattice parameter deviations (predicted − experimental), Δ*E* and RMSD_15_ for the experimental and predicted structures of molecule (XVII) α = γ = 90° in all structures.

	Rank	Δ*E*† (kJ mol^−1^)	Density (g cm^−3^)	*a* (Å)	*b* (Å)	*c* (Å)	β (°)	RMSD_15_‡ (Å)
Expt. (*T* = 174 K)	–	–	1.837	12.639 (1)	5.979 (1)	11.422 (1)	96.807 (1)	−
								
Predicted amongst first three								
Neumann, Leusen, Kendrick, van de Streek	1	−1.64[Table-fn tfn10]	+0.7%	0.0%	−1.0%	+0.5%	−0.1%	0.045
Price, Habgood	2	+1.05	−0.3%	+0.2%	−2.0%	+1.6%	−0.4%	0.130
								
Present in list, outside of first three predictions								
Day	4	+3.24	−0.2%	−0.2%	−2.6%	+2.7%	−2.0%	0.191
van Eijck	6	+3.67	−1.5%	+1.0%	−0.8%	+1.2%	−0.4%	0.102
Desiraju, Thakur, Tiwari, Pal	65	+5.00	+5.3%	+1.4%	−2.3%	−4.2%	−0.1%	0.264
Boerrigter	121	+6.39	−0.9%	+2.8%	−4.3%	+2.9%	+1.0%	0.270

† Δ*E* is calculated with respect to the lowest energy structure predicted by the same research group.

‡ RMSD_15_ is calculated using a 15 molecule comparison in the Materials Module of *Mercury*, ignoring H atoms.

§ Δ*E* for the global minimum is calculated with respect to the second lowest energy structure.

**Table 5:** Lattice parameter deviations (predicted − experimental), Δ*E* and RMSD for the experimental and predicted structures of molecule (XVIII) α = β = γ = 90° in all structures.

	Rank	Δ*E*† (kJ mol^−1^)	Density (g cm^−3^)	*a* (Å)	*b* (Å)	*c* (Å)	RMSD_15_‡ (Å)
Expt. (*T* = 174 K)	–	–	1.566	9.889 (1)	8.887 (1)	24.969 (3)	–
							
Predicted amongst first three							
Neumann, Leusen, Kendrick, van de Streek	1	−1.30§	−1.2%	+0.4%	−1.0%	+1.9%	0.122
							
Present in list, outside of first three predictions							
Orendt, Grillo, Ferraro, Facelli	4	+2.53	+3.9%	+1.0%	−1.5%	−3.3%	0.252
Desiraju, Thakur, Tiwari, Pal	13	+5.92	−7.7%	+4.4%	+0.6%	+0.7%	0.362
Scheraga, Arnautova	29	+8.21	−5.2%	−0.1%	+6.2%	−0.6%	0.390

† Δ*E* is calculated with respect to the lowest energy structure predicted by the same research group.

‡ RMSD_15_ is calculated using a 15 molecule comparison in the Materials Module of *Mercury*, ignoring H atoms.

§ Δ*E* for the global minimum is calculated with respect to the second lowest energy structure.

**Table 6:** Lattice parameter deviations (predicted − experimental), Δ*E* and RMSD for the experimental and predicted structures of molecular salt (XIX) α = β = γ = 90° in all structures.

	Rank	Δ*E*† (kJ mol^−1^)	Density (g cm^−3^)	*a* (Å)	*b* (Å)	*c* (Å)	RMSD_15_‡ (Å)
Expt. (*T* = 200 K)	–	–	1.481	23.501 (3)	3.714 (1)	12.654 (1)	–
							
Predicted amongst first three							
van Eijck	2	+0.83	−2.2%	+1.9%	−0.4%	+0.7%	0.220
Neumann, Leusen, Kendrick, van de Streek	3	+6.73	+0.5%	+0.6%	+1.1%	−2.2%	0.151
							
Present in list, outside of first three predictions							
Boerrigter	12	+2.47	−8.2%	+4.0%	−0.1%	+4.8%	0.367
Day, Cruz-Cabeza	27	+12.62	−1.6%	+4.4%	+2.0%	+1.6%	0.209

† Δ*E* is calculated with respect to the lowest energy structure predicted by the same research group.

‡ RMSD_15_ is calculated using a 15 molecule comparison in the Materials Module of *Mercury*, ignoring H atoms.

**Table 7:** Lattice parameter deviations (predicted − experimental), Δ*E* and RMSD_15_ for the experimental and predicted structures of molecule (XX) α = γ = 90° in all structures.

	Rank	Δ*E*† (kJ mol^−1^)	Density (g cm^−3^)	*a* (Å)	*b* (Å)	*c* (Å)	β (°)	RMSD_15_‡ (Å)
Expt. (*T* = 150 K)	–	–	1.411	14.078 (1)	6.356 (1)	25.310 (2)	96.063 (2)	–
								
Predicted amongst first three								
Day, Cruz-Cabeza	1	−0.53§	−2.4%	+0.3%	−1.8%	+3.9%	−0.4%	0.429
Price, Kazantsev, Karamertzanis, Adjiman, Pantelides	1	−0.78§	–0.6%	+1.3%	−0.6%	+0.2%	+1.3%	0.178
								
Present in list, outside of first three predictions								
Neumann, Leusen, Kendrick, van de Streek	7	+1.90	+0.1%	+0.6%	−0.9%	+0.2%	−0.7%	0.113

† Δ*E* is calculated with respect to the lowest energy structure predicted by the same research group.

‡ RMSD_15_ is calculated using a 15 molecule comparison in the Materials Module of *Mercury*, ignoring H atoms.

§ Δ*E* for the global minimum is calculated with respect to the second lowest energy structure.

**Table:** α = γ = 90° in all structures.

	Rank	Δ*E*† (kJ mol^−1^)	Density (g cm^−3^)	*a* (Å)	*b* (Å)	*c* (Å)	β (°)	RMSD_15_[Table-fn tfn20] (Å)
Expt. (*T* = 150 K)	–	–	1.639	9.790 (7)	3.609 (3)	21.583 (16)	91.462 (14)	–
								
Present in list, outside of first three predictions								
Day	12	+2.96	+2.4%	−2.9%	−0.4%	+1.1%	+0.3%	0.159
van Eijck	29	+12.47	+10.1%	−4.9%	−2.4%	−2.5%	+0.8%	0.208
Neumann, Leusen, Kendrick, van de Streek	81	+8.85	+2.0%	−2.8%	+1.2%	−0.4%	−0.5%	0.228
Price, Braun	117	+12.69	+3.7%	−1.5%	−1.9%	−0.1%	+0.8%	0.108

**(b):** Lattice parameter deviations (predicted − experimental), Δ*E* and RMSD_15_ for the predicted structures of hydrate (XXI) with alternative H-atom placement to the experimental structure. α = γ = 90° in all structures.

	Rank	Δ*E*[Table-fn tfn21] (kJ mol^−1^)	Density (g cm^−3^)	*a* (Å)	*b* (Å)	*c* (Å)	β (°)	RMSD_15_[Table-fn tfn22] (Å)
Expt. (*T* = 150 K)	–	–	1.639	9.790 (7)	3.609 (3)	21.583 (16)	91.462 (14)	–
								
Predicted amongst first three								
Van Eijck	1	−2.43[Table-fn tfn23]	+13.1%	−5.4%	−3.8%	−2.8%	+1.2%	0.232
Price, Braun	3	+1.08	+5.8%	−1.0%	−4.0%	−0.6%	+0.1%	0.224
								
Present in list, outside of first three predictions								
Desiraju, Thakur, Tiwari, Pal	11	+0.19	+6.2%	+0.6%	−3.3%	−3.2%	−0.4%	0.642
Day	61	+6.96	+4.5%	−1.0%	−1.4%	−2.1%	−1.6%	0.218
Neumann, Leusen, Kendrick, van de Streek	174	+11.30	+2.9%	−2.7%	−0.2%	+0.1%	−0.4%	0.192

**(c):** Lattice parameter deviations (predicted − experimental), Δ*E* and RMSD_15_ for the experimental and predicted structures of KONTIQ [form (1)]. α = γ = 90° in all structures.

	Rank	Δ*E*[Table-fn tfn24] (kJ mol^−1^)	Density (g cm^−3^)	*a* (Å)	*b* (Å)	*c* (Å)	β (°)	RMSD_15_† (Å)
Expt. (*T* = 150 K)	–	–	1.599	5.794 (4)	4.719 (5)	28.688 (5)	95.080 (30)	–
								
Present in list								
Neumann, Leusen, Kendrick, van de Streek	19	+0.22	+3.6%	+1.3%	−5.4%	+0.8%	+0.6%	0.211
Day	48	+6.61	+3.1%	+4.3%	−6.3%	+0.2%	−0.6%	0.295
van Eijck	74	+18.80	+10.8%	+6.1%	−17.0%	+2.2%	−5.0%	0.690
Desiraju, Thakur, Tiwari, Pal	143	+12.11	+7.6%	−4.4%	−3.3%	+1.1%	+2.9%	0.442
Boerrigter	282	+13.18	−2.6%	−8.42%	+10.2%	+2.8%	+4.7%	0.631
Price, Braun	338	+11.87	+1.9%	+11.0%	−14.6%	+5.0%	+6.8%	0.683

**(d):** Lattice parameter deviations (predicted − experimental), Δ*E* and RMSD_15_ for the experimental and predicted structures of KONTIQ01 [form (2)]. α = γ = 90° in all structures.

	Rank	Δ*E*‡ (kJ mol^−1^)	Density (g cm^−3^)	*a* (Å)	*b* (Å)	*c* (Å)	β (°)	RMSD_15_§ (Å)
Expt. (*T* = 150 K)	–	–	1.636	14.150 (10)	3.622 (9)	15.028 (10)	97.520 (70)	–
								
Present in list								
van Eijck	9	+8.44	+11.0%	−2.2%	−2.7%	−5.2%	+0.5%	0.206
Price, Braun	23	+4.83	+3.9%	+0.7%	−3.6%	−0.6%	+0.5%	0.186
Neumann, Leusen, Kendrick, van de Streek	49	+0.34	+2.0%	−1.0%	−0.1%	−2.2%	+0.1%	0.090
Day	53	+6.68	+4.7%	−1.4%	−0.6%	−2.1%	+1.7%	0.135
Desiraju, Thakur, Tiwari, Pal	126	+10.93	+4.9%	1.4%	−2.1%	−0.7%	+2.2%	0.228

† Δ*E* is calculated with respect to the lowest energy structure predicted by the same research group.

‡ RMSD_15_ is calculated using a 15 molecule comparison in the Materials Module of *Mercury*, ignoring H atoms.

§ Δ*E* is calculated with respect to the lowest energy structure predicted by the same research group.

¶ RMSD_15_ is calculated using a 15 molecule comparison in the Materials Module of *Mercury*, ignoring H atoms.

†† Δ*E* for the global minimum is calculated with respect to the second lowest energy structure.

‡‡ Δ*E* is calculated with respect to the lowest energy structure predicted by the same research group.

§§ RMSD_15_ is calculated using a 15 molecule comparison in the Materials Module of *Mercury*, ignoring H atoms.

¶¶ Δ*E* is calculated with respect to the lowest energy structure predicted by the same research group.

††† RMSD_15_ is calculated using a 15 molecule comparison in the Materials Module of *Mercury*, ignoring H atoms.

**Table 9:** Summary of computational resources used by some of the participants in CSP2010

Group	Comments on computing time used	Total computational cost, approximately normalized to 3.0 GHz CPU hours †
Boerrigter	All calculations were performed on an Intel core i7-950 (3.07 GHz) (single core). Approximate execution times:	∼ 3800 CPU hours
	(XVI): 90 h	
	(XVII): 100 h	
	(XVIII): 350 h	
	(XIX): 650 h	
	(XX): 2105 h	
	(XXI): 600 h	
Day, Cruz-Cabeza	Most calculations were performed on AMD Opteron 280, 2.6 GHz processors, although parts of the calculations were performed on CPUs with lower performance.	∼ 91 400 CPU hours
	(XVI): 110 h	
	(XVII): 1941 h	
	(XVIII): 21 051 h	
	(XIX): 6097 h	
	(XX): 54 090 h	
	(XXI) 22 197 h	
Desiraju, Thakur, Tiwari, Pal	Calculations were performed on four 3.2 GHz processors.	∼ 4600 CPU hours
	(XVI): 114 h	
	(XVII): 2303 h	
	(XVIII): 324 h	
	(XIX): 114 h	
	(XXI): 1431 h	
Hofmann	Calculations were performed on 3.0 GHz processors.	∼ 1600 CPU hours
	(XVI): 2 h	
	(XVII): 7 h	
	(XVIII): 12 h	
	(XIX): 694 h	
	(XX): 670 h	
	(XXI): 187 h‡	
Neumann, Leusen, Kendrick, van de Streek	Approximately 122 000 CPU hours on 2.8 GHz processors, mostly spent on the generation of reference data for force field parameterization and the final energy ranking with the hybrid method	∼ 115 000 CPU hours
Price *et al.*	(XVI): 200 h	∼ 195 000 CPU hours
	(XVII): 5000 h	
	(XVIII): 14 000 h	
	(XIX): 3000 h	
	(XX): 120 000 h	
	(XXI): 52 800 h	
Van Eijck	Calculations performed on 2.66 GHz processors. Molecular calculations: 27 h Structure generation: 4910 h energy minimization: 4526 h	∼ 9500 CPU hours
Della Valle, Venuti	Approximately 4400 h on 2.2 GHz processors. Initial rigid-molecule optimizations, DFT calculations, potential fitting and final flexible-molecule optimizations consumed 40, 11, 26 and 22% of the time.	∼ 3200 CPU hours
Maleev, Zhitkov	Crystal structure search + energy minimization 2 intel^®^ core™ i5cpu 750 at 2.67 GHz processors and each has 2 GB of memory	∼ 7500 CPU hours
Misquitta, Pickard & Needs	AIRSS/DFT-D search: 130 000 core hours First search using structures obtained with FIT+Q potential: 30 000 core hours Post-Blind test analysis: < 2000 core hours	∼ 162 000 CPU hours
Scheraga, Arnautova	Calculations were carried out on Intel Xeon 2.4 GHz processors	∼ 1300 CPU hours
	(XVI:) 150 h	
	(XVII): 150 h	
	(XVIII): 610 h	
	(XIX): 720 h	

† Note that the large difference in total CPU time presented here is in part due to the various participants electing to predict differing numbers of target molecules and also represents the use of large parallelized computing arrays.

‡ For compound (XXI) the calculation was interrupted after 187 h due to not being able to estimate the convergence in the energetic landscape by that time.

## Footnotes

1 Supplementary data for this paper are available from the IUCr electronic archives (Reference: BK5106). Services for accessing these data are described at the back of the journal.
