# Supplementary material for: Towards crystal structure prediction of complex organic compounds – a report on the fifth blind test
Source: Acta Crystallogr B. 2011 Nov 17;67(Pt 6):535–51. doi: 10.1107/S0108768111042868 (PMC3222142; doi:10.1107/S0108768111042868)
Supplement: Supplementary file 4 [file b-67-00535-sup4.pdf]

Supplementary Data: Details of Predictions located during post-analysis

Table S3. Lattice parameter deviations, Δ*E* and RMSD for the experimental and predicted structures of molecule XVI. α = β = γ = 90° in all structures.

	Rank	Δ*E* [a] (kJ/mol)	density (g/cm^3)	*a* (Å)	*b* (Å)	*c* (Å)	$RMSD_{15}$ [b] (Å)
expt. (*T* = 174 K)	-	-	1.385	9.645(2)	7.381(1)	16.185(3)	-
**not located in search, but energy minimised in post-analysis**							
Hofmann	-	+1.51	-2.5%	+2.5%	+0.6%	-0.6%	0.115
Misquitta, Pickard, Needs	-	-0.05[c]	+5.6%	-0.4%	-3.9%	-1.1%	0.145
Scheraga, Arnautova	-	+2.59	-5.9%	+1.6%	+3.9%	+0.6%	0.224

[a] Δ*E* is calculated with respect to the lowest energy structure predicted by the same research group.
[b] $RMSD_{15}$ is calculated using a 15 molecule comparison in the Materials Module of Mercury, ignoring H atoms.
[c] Δ*E* for the global minimum is calculated with respect to the second lowest energy structure.

Table S4. Lattice parameter deviations, Δ*E* and $RMSD_{15}$ for the experimental and predicted structures of molecule XVII. α = γ = 90° in all structures.

	Rank	Δ*E* [a] (kJ/mol)	density (g/cm^3)	*a* (Å)	*b* (Å)	*c* (Å)	β (°)	$RMSD_{15}$ [b] (Å)
expt. (*T* = 174 K)	-	-	1.837	12.639(1)	5.979(1)	11.422(1)	96.807(1)	-
**not located in search, but energy minimised in post-analysis**								
Hofmann	-	+2.75	-1.8%	+0.9%	-0.6%	+1.8%	+2.8%	0.229
Scheraga, Arnautova	-	+5.34	-2.6%	+2.1%	0.0%	+0.2%	+0.6%	0.193

[a] Δ*E* is calculated with respect to the lowest energy structure predicted by the same research group.
[b] $RMSD_{15}$ is calculated using a 15 molecule comparison in the Materials Module of Mercury, ignoring H atoms.

Table S5. Lattice parameter deviations, Δ*E* and RMSD for the experimental and predicted structures of molecule XVIII. α = β = γ = 90° in all structures.

	Rank	Δ*E* [a] (kJ/mol)	density (g/cm^3)	*a* (Å)	*b* (Å)	*c* (Å)	$RMSD_{15}$ [b] (Å)
expt. (*T* = 174 K)	-	-	1.566	9.889(1)	8.887(1)	24.969(3)	-
**not located in search, but energy minimised in post-analysis**							
Hofmann	-	+2.56	-1.6%	+1.6%	+0.2%	+0.2%	0.135
Price, Price	-	+5.03	-2.9%	+0.7%	+1.9%	0.0%	0.100
Boerrigter	-	-0.58 [c]	+0.3%	+1.7%	+0.5%	-2.0%	0.439

Van Eijck	-	+29.30	-1.7%	+0.8%	+2.4%	-0.3%	0.188

[a] ΔE is calculated with respect to the lowest energy structure predicted by the same research group.
[b] $RMSD_{15}$ is calculated using a 15 molecule comparison in the Materials Module of Mercury, ignoring H atoms.
[c] ΔE for the global minimum is calculated with respect to the second lowest energy structure.

Table S6. Lattice parameter deviations, ΔE and RMSD for the experimental and predicted structures of molecular salt XIX. $\alpha = \beta = \gamma = 90°$ in all structures.

	Rank	ΔE [a] (kJ/mol)	density (g/cm^3)	*a* (Å)	*b* (Å)	*c* (Å)	$RMSD_{15}$ [b] (Å)
expt. (T = 200 K)	-	-	1.481	23.501(3)	3.714(1)	12.654(1)	-
**not located in search, but energy minimised in post-analysis**							
Hofmann	-	+12.43	-7.3%	+6.0%	+0.3%	+0.7%	0.301
Price, Mohamed	-	+10.21	-3.2%	-2.2%	+2.0%	+3.7%	0.265
Scheraga, Arnautova	-	+0.32	-1.4%	-2.4%	-1.2%	+5.1%	0.542

[a] ΔE is calculated with respect to the lowest energy structure predicted by the same research group.
[b] $RMSD_{15}$ is calculated using a 15 molecule comparison in the Materials Module of Mercury, ignoring H atoms.

Table S7. Lattice parameter deviations, ΔE and $RMSD_{15}$ for the experimental and predicted structures of molecule XX. $\alpha = \gamma = 90°$ in all structures.

	Rank	ΔE [a] (kJ/mol)	density (g/cm^3)	*a* (Å)	*b* (Å)	*c* (Å)	β (°)	$RMSD_{15}$ [b] (Å)
expt. (T = 150 K)	-	-	1.411	14.078(1)	6.356(1)	25.310(2)	96.063(2)	-
**not located in search, but energy minimised in post-analysis**								
Hofmann	-	+2.43	+0.4%	+1.0%	-2.1%	+0.9%	+1.3%	0.297
Van Eijck	-	-11.40[c]	-0.8%	+1.4%	-2.5%	+2.6%	+4.1%	0.435

[a] ΔE is calculated with respect to the lowest energy structure predicted by the same research group.
[b] $RMSD_{15}$ is calculated using a 15 molecule comparison in the Materials Module of Mercury, ignoring H atoms.
[c] ΔE for the global minimum is calculated with respect to the second lowest energy structure.

Table S8a. Lattice parameter deviations, ΔE and $RMSD_{15}$ for the experimental and predicted structures of hydrate XXI (with matching hydrogen placement). $\alpha = \gamma = 90°$ in all structures.

	Rank	ΔE [a] (kJ/mol)	density (g/cm^3)	*a* (Å)	*b* (Å)	*c* (Å)	β (°)	$RMSD_{15}$ [b] (Å)
expt. (T = 150 K)	-	-	1.639	9.790(7)	3.609(3)	21.583(16)	91.462(14)	-
**not located in search, but energy minimised in post-analysis**								
Hofmann	-	+2.10	+1.9%	+1.3%	-1.5%	+2.7%	+2.7%	0.159

[a] ΔE is calculated with respect to the lowest energy structure predicted by the same research group.
[b] $RMSD_{15}$ is calculated using a 15 molecule comparison in the Materials Module of Mercury, ignoring H atoms.

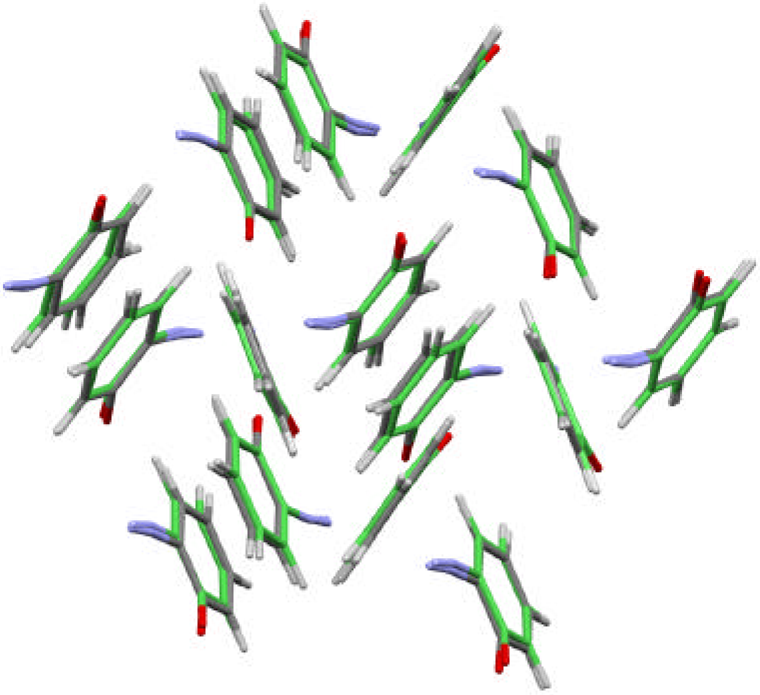

Figure S1. Overlay of the unit-cell contents of the observed crystal structure XVI (green) and Neumann *et al.* XVI.1 (grey). RMSD$_{15}$ 0.157 Å

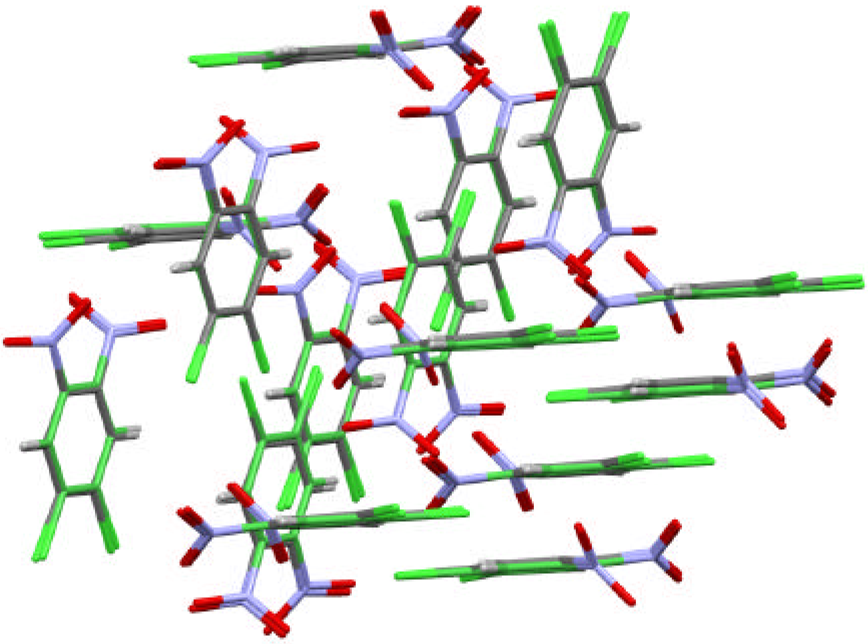

Figure S2. Overlay of the unit-cell contents of the observed crystal structure XVII (green) and Price *et al.* XVII.2 (grey). RMSD$_{15}$ 0.130 Å

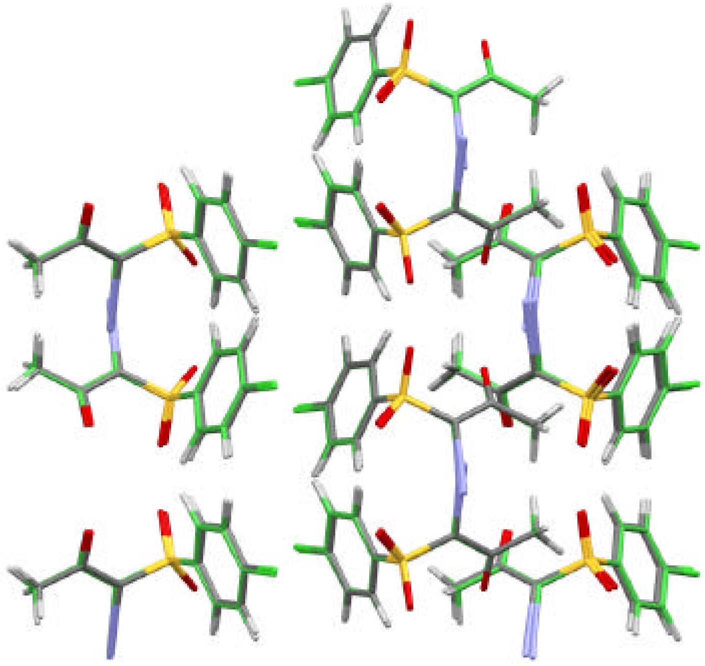

Figure S3. Overlay of the unit-cell contents of the observed crystal structure XVIII (green) and Neumann *et al.* XVIII.1 (grey). RMSD 0.122 Å

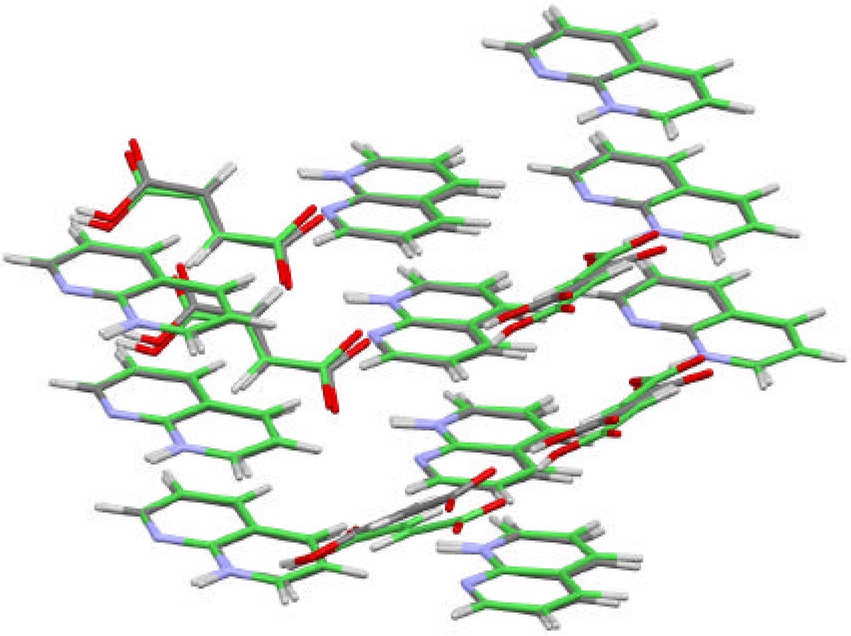

Figure S4. Overlay of the unit-cell contents of the observed crystal structure XIX (green) and van Eijck XIX.2 (grey). RMSD 0.220 Å

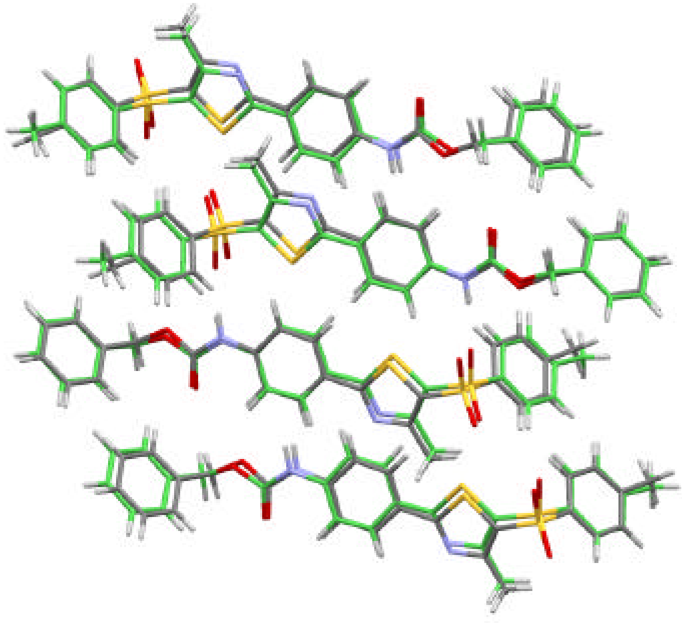

Figure S5. Overlay of the unit-cell contents of the observed crystal structure XX (green) and Day *et al.* XX.1 (grey). RMSD 0.429 Å
